# Supplementary material for: HIVprotI: an integrated web based platform for prediction and design of HIV proteins inhibitors
Source: J Cheminform. 2018 Mar 9;10:12. doi: 10.1186/s13321-018-0266-y (PMC5845081; doi:10.1186/s13321-018-0266-y)
Supplement: Supplementary file 2 — Additional file 2. Source code of HIVProtI web server. [file 13321_2018_266_MOESM2_ESM.zip › HIVprotI_Source-code/hivprotI/weka-3-6-6/doc/weka/gui/arffviewer/package-summary.html]

weka.gui.arffviewer


---


|  |  |  |
| --- | --- | --- |
| **Overview** \| **Package** \| Class \| **Tree** \| **Deprecated** \| **Index** \| **Help** \| **Weka's home** | |  |
| **PREV PACKAGE**   **NEXT PACKAGE** | **FRAMES**    **NO FRAMES**     **All Classes** |  |


---

## Package weka.gui.arffviewer

| **Class Summary** | |
| --- | --- |
| **ArffPanel** | A Panel representing an ARFF-Table and the associated filename. |
| **ArffSortedTableModel** | A sorter for the ARFF-Viewer - necessary because of the custom CellRenderer. |
| **ArffTable** | A specialized JTable for the Arff-Viewer. |
| **ArffTableCellRenderer** | Handles the background colors for missing values differently than the DefaultTableCellRenderer. |
| **ArffTableModel** | The model for the Arff-Viewer. |
| **ArffViewer** | A little tool for viewing ARFF files. |
| **ArffViewerMainPanel** | The main panel of the ArffViewer. |
| **Messages** | Messages. |

---


|  |  |  |
| --- | --- | --- |
| **Overview** \| **Package** \| Class \| **Tree** \| **Deprecated** \| **Index** \| **Help** \| **Weka's home** | |  |
| **PREV PACKAGE**   **NEXT PACKAGE** | **FRAMES**    **NO FRAMES**     **All Classes** |  |


---
